# Unveiling Intermittency in the Control of Quiet Upright Standing: Beyond Automatic Behavior

**DOI:** 10.3389/fneur.2018.00850

**Published:** 2018-10-10

**Authors:** John F. Stins, Melvyn Roerdink

**Affiliations:** Department of Human Movement Sciences, Faculty of Behavioural and Movement Sciences, Vrije Universiteit Amsterdam, Amsterdam Movement Sciences, Amsterdam, Netherlands

**Keywords:** postural control, intermittency, dual-tasking, postural sway, attention, reaction time, sway density curve

## Abstract

The control of posture, as in quiet upright standing, is distributed among postural reflexes and higher (cortical) centers. According to the theory of “intermittent control,” the control of posture involves a rapid succession of brief periods of postural stability, during which the body dwells relatively motionless in a particular posture, and postural instability, during which the body rapidly transits to a new stable point. This theory assumes a combination of stiffness control, keeping the body in the same position, and top-down ballistic control, moving the body to a new reference position. We tested the prediction that exerting ballistic control consumes more attention, relative to stiffness control, using variations in reaction time as our index of attention load. Slower reactions to external stimulus events were expected if these events happen to coincide with ballistic control regimes compared to stiffness regimes, as unveiled from local features of the posturogram. Thirty-two participants stood on a force plate, and were instructed to press a hand-held button as soon as they heard a stimulus tone. About 40 stimuli were presented at random instances during a 3-min trial. Postural control regimes were characterized using sway-density analysis for each stimulus-response interval, by computing local dwell times from the corresponding center-of-pressure samples. We correlated stimulus-response durations with the corresponding local dwell times, and also with local velocity and local eccentricity (distance from the origin). As predicted, an overall negative correlation was observed, meaning that shorter dwell times are associated with longer stimulus-response intervals, as well as a positive correlation with local center-of-pressure velocity. The correlation between reaction times and local eccentricity was not significant. Thus, by mapping stimulus-response intervals to local center-of-pressure features we demonstrated attentional fluctuations in the control of quiet upright standing, thereby validating a core assumption underlying the notion of intermittent postural control.

## Introduction

Body sway during quiet upright standing reflects attempts of the actor to control the unstable “inverted pendulum,” i.e., the erect human body. In theory, it would be possible to apply a constant mechanical stiffness of the muscle-tendon complex acting around the ankle joint. If stiffness is sufficiently high, then the inverted pendulum will stay in place. However, empirical measurements performed by Loram and Lakie (1) showed that ankle stiffness was lower than the gravitational toppling torque, implying that an additional mechanism was required to maintain quiet upright standing.

This mechanism arguably involved serial ballistic, neurally generated, torques that “kick” the body center of mass in a particular direction. This form of postural control has been labeled intermittent or “saccadic” control [e.g., (2)]. An important characteristic of intermittent control is its anticipatory nature. Phasic neural commands, sent out to the muscles, generate anticipatory torques based on a prediction of imminent destabilization, and bring the center of mass back to a stable state. Intermittent control thus implies a neural representation of the “inverted pendulum” dynamics that predicts the postural consequences of phasic applied torques.

Is it possible to identify such intermittent or discrete instances of control in the trajectory of the center of pressure (COP) during an actual quiet standing episode? Yes, according to an inventive sway-density analysis method proposed by Baratto et al. (2), and adopted by others since then [e.g., (3–5)]. This method involves constructing so-called sway-density curves, unveiling instances when the COP is relatively stationary or transient (see section Materials and Methods for details). Briefly, the approach assumes a “waxing and waning” between episodes wherein the center of mass (and the COP) is relatively motionless (i.e., high density, long dwell times) and episodes wherein the COP quickly shifts position (i.e., low density, short dwell times). This process has been likened to sequences of saccades and fixations, which alternate and repeat in quick succession (3). During the “fixations” the COP shows little activity, likely representing the contribution of ankle stiffness. On the other hand, the “saccades” involve quick displacements of the COP, likely representing phasic neural commands generating anticipatory torques to bring the erect body back to a stable state, which is then again followed by a period of postural inactivity, ad infinitum.

In a recent paper, Villarrasa-Sapiña et al. (4) described intermittent control as consisting of two control mechanisms; one involving the mechanical properties of the ankle muscles (a.k.a. intrinsic feedback, or “passive control”), and one involving anticipatory activation of the muscles (feedforward control, or “active control”). It is assumed that episodes in the posturogram with short dwell times represent anticipatory top-down balance control that drive the COP (and hence the center of mass) back to a stable state (4). According to Baratto et al. (2), these episodes represent complex sensory processing, for example estimating a stable future (intended) state of the center-of-mass. The sway-density analysis has been successfully applied to demonstrate that the sensory regulation of postural control is affected in individuals with idiopathic scoliosis (6) and in individuals with obesity (7, 4).

Despite the promising empirical and theoretical embedding of this analysis [but see (5)], there has been no independent test of one of its core assumptions, namely that during quiet standing there is a quick back-and-forth of two control regimes; the passive feedback control and active feedforward control, as outlined above. In this paper we argue that the two control strategies likely differ in their computational and attentional complexity. More specifically, feedforward control is applied by the central nervous system based on an internal model of the body dynamics, which likely comes with an associated computational cost. In contrast, stiffness control is peripheral by nature and is likely not—or considerably less—computationally demanding. If this holds, then it should be possible to observe differences in computational cost using a concurrent stimulus-response reaction-time task, thereby taxing the differential attentional demands of the respective control modes, represented by specific local posturographic state variables (i.e., position, velocity, dwell times).

To this end, we first briefly describe a study by Teasdale et al. (8) which served as inspiration for our current study. That study asked whether young and older (otherwise healthy) participants differed in their attentional requirements to maintain static balance. During the quiet standing task, participants heard a tone at unpredictable instances upon which they had to press a handheld button as quickly as possible. Stimulus-response reaction times (RT) served as an index of the attentional requirements needed to perform the task, i.e., upright standing while responding to the tone. Teasdale et al. (8) reasoned that if the COP happened to be in a more eccentric position (relative to the origin of the posturogram), posture was presumably less stable, requiring deployment of attentional resources to bring the COP toward a more central (and putatively more stable) position. Thus, if an auditory stimulus happened to coincide with a more eccentric COP position, then—following Teasdale et al.'s (8) reasoning—this should give rise to longer RTs. This was indeed the case, but only for the group of older participants (8).

Teasdale et al. (8) focused exclusively on postural eccentricity. However, they did not consider the possibility that episodes of relatively stationary and transient COP excursions may occur anywhere in the posturogram, that is, at eccentric and central positions alike. As argued above, some episodes involve rapid, self-generated anticipatory COP displacements and likely reflect attention-demanding postural computations, whereas other episodes are relatively stationary and not (or less) computationally demanding. We adopted the experimental paradigm developed by Teasdale et al. (8) to test the hypothesis that the attentional requirements of postural control “wax and wane” during quiet upright standing, with greater requirements for active compared to passive control regimes. We used stimulus-response RTs as an index for the required attentional involvement (higher RTs represent greater attentional requirements) and related them to local posturographic state features like local COP eccentricity, local COP velocity and local dwell times derived from sway-density curves. Local COP eccentricity was included to replicate Teasdale et al.'s (8) findings. Local COP velocity and local dwell times were included to unveil passive and active control episodes. Episodes in which the COP is relatively stationary are characterized by low velocity and high dwell times, which are assumed to reflect episodes of passive control. Vice versa, COP episodes with high velocity and low dwell times are assumed to reflect episodes of active control. By correlating RT values to local COP velocities and local dwell times, we could evaluate the hypothesis that the attentional requirement for controlling upright quiet stance fluctuates depending on the relative contribution of the two control regimes. More precisely, we predict (a) an overall positive association between RT and local COP velocity and (b) an overall negative association between RT and local dwell times.

## Materials and methods

### Participants

We tested 32 healthy young participants (15 females and 17 males). Their mean (±SD) age was 21.7 (±2.1) years. The study was approved by the local ethics committee and informed consent was obtained from all participants.

### Procedure

Participants were instructed to stand still on a force plate (1 × 1 m, custom made) and to pay attention to a sequence of tones that was presented over computer loud speakers, positioned behind the participant at a distance of 1.5 m. Tones (8 kHz) were presented at random intervals ranging between 2 and 6 s. Participants had to press a small response key that was held in the right hand, as soon as they heard the tone. The computer sampled the stimulus tones, the response events, and the anterior-posterior (AP) and mediolateral (ML) COP data at 1 kHz.

The experiment was divided into four trials; (1) standing on a firm surface (i.e., the metal surface of the force plate), (2) standing on a piece of foam (40 × 40 × 8 cm, medium density) laying atop the force plate, (3) standing on a firm surface again, and (4) standing again on foam. Each trial lasted 181 s, thus yielding 181.000 data samples per trial. During each trial there were about 40 stimuli. Between trials there was a break of ~1 min, during which the experimenter attached or removed the piece of foam.

### Data analysis

#### Stimulus-response reaction times

We had to exclude three participants from the analyses because of a measurement error (i.e., failure to record the responses). For the 29 remaining participants, we ensured that the recorded stimulus-response pairs were valid by (1) excluding responses faster than 100 ms, (2) excluding responses slower than 600 ms, and (3) by excluding stimuli without a recorded response. Two participants exhibited RTs that were considerably slower than the rest with responses lasting well over 600 ms on many occasions. We decided to discard these participants from further analyses. For the remaining 27 participants (our final sample) we found that (1) there were never responses below 100 ms, (2) there were twelve responses slower than 600 ms, and (3) there was one stimulus without a recorded response. These events were discarded from further analyses. Furthermore, we discarded stimulus-response pairs falling in the first 5 or final 5 s of a trial to prevent the influence of starting or stopping the trials. From the remaining 4,515 valid stimulus-response pairs (yielding on average 42 valid pairs per trial, range 37–44), the mean stimulus-response reaction-time interval was determined, separately for each trial.

#### Global posturographic outcomes: eccentricity, velocity, and dwell time

AP and ML COP time series were filtered with a bi-directional, second-order low-pass Butterworth filter with a cut-off frequency of 12.5 Hz. We then removed the first and last 5 s of each trial (see above). For each trial, the mean eccentricity (defined as mean distance to the origin of the posturogram, in mm) and the mean sway velocity (traveled distance in the posturogram per unit time, in mm/s) were determined. Greater eccentricity and velocity were expected for standing on foam. Dwell times were determined from sway-density curves derived by counting, for each sample *i*, the number of consecutive samples on the posturogram falling inside a circle of given radius *R*, yielding, for each sample *i*, the duration in ms that the COP remained inside that circle [see, for example, (4) for a graphical illustration of the method]. These durations, also known as dwell times, critically depend on the overall magnitude of the posturogram (i.e., shorter durations for larger posturograms, thus supposedly shorter dwell times for foam than firm surfaces) and the size of the radius *R* (i.e., longer dwell times for larger radii). Although not the main topic of our research, we decided to investigate the effect of choice of radius on overall dwell times. The default setting for the radius in the literature is 2.5 mm [e.g., (2)], but some studies [e.g., (9)] have manipulated the size of *R* and examined its effect on the number of peaks and associated heights (i.e., peak dwell times) of the sway density curve. Since we were particularly interested in episodes at a specific time scale, namely episodes similar to the stimulus-response reaction times, we computed sway-density curves for ten different radii (0.25, 0.50, …, 2.50 mm), from which the mean dwell times were determined. The radius yielding dwell times similar to the stimulus-response durations will be used for the subsequent relational analyses, as described next.

#### Local posturopgraphic outcomes and their relation to stimulus-response reaction times

**Figure 1:**
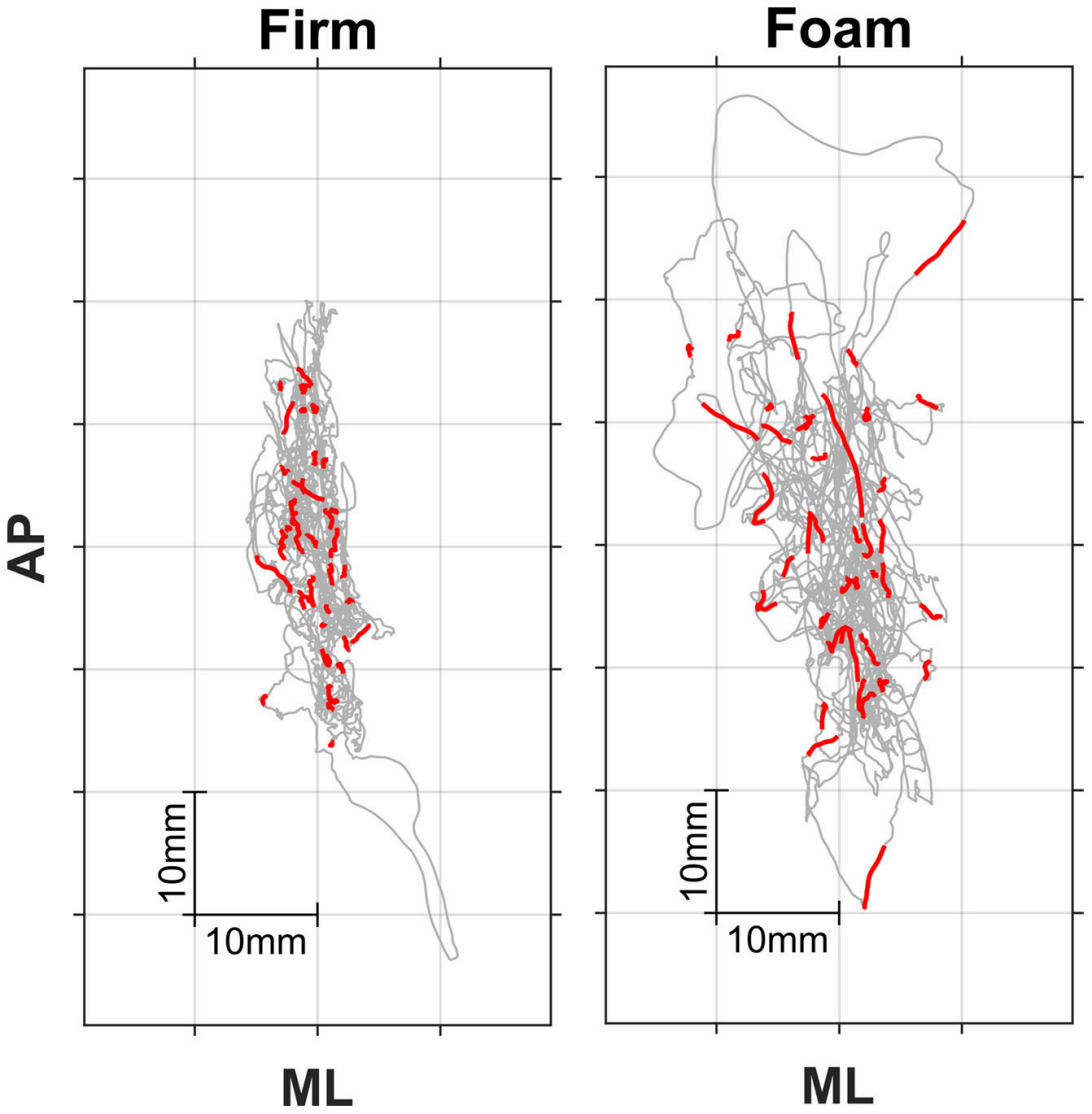
Posturograms for firm **(Left)** and foam **(Right)** surface conditions. Trials lasted 181 s. Anterior-posterior (AP) vs. mediolateral (ML) center-of-pressure trajectories are depicted in gray, while superimposed red traces represent the 42 or so stimulus-response episodes during a trial.

Figure 1 shows two individual posturograms (firm and foam), in which the red traces represent segments in the posturogram corresponding to the stimulus-response intervals. Our main interest was in the relationship between local posturographic outcomes (i.e., computed over segments in the posturogram corresponding to the stimulus-response intervals, i.e., the red traces in Figure 1) and stimulus-response reaction times, because this may reveal whether certain episodes within the posturogram have heightened attentional costs. To this end, we determined eccentricity, velocity and dwell times locally from COP episodes corresponding to individual stimulus-response intervals. If, for example, a given stimulus-response pair had a RT value of 200 ms, we used the posturographic data spanning this interval to determine local eccentricity, velocity and dwell times (thus based on 200 posturographic samples, starting with the sample corresponding to stimulus onset and ending with the sample corresponding to key press). We did this for each valid stimulus-response pair (see above), resulting in 37–44 values for local eccentricity, velocity, and dwell time per trial.

**Figure 2:**
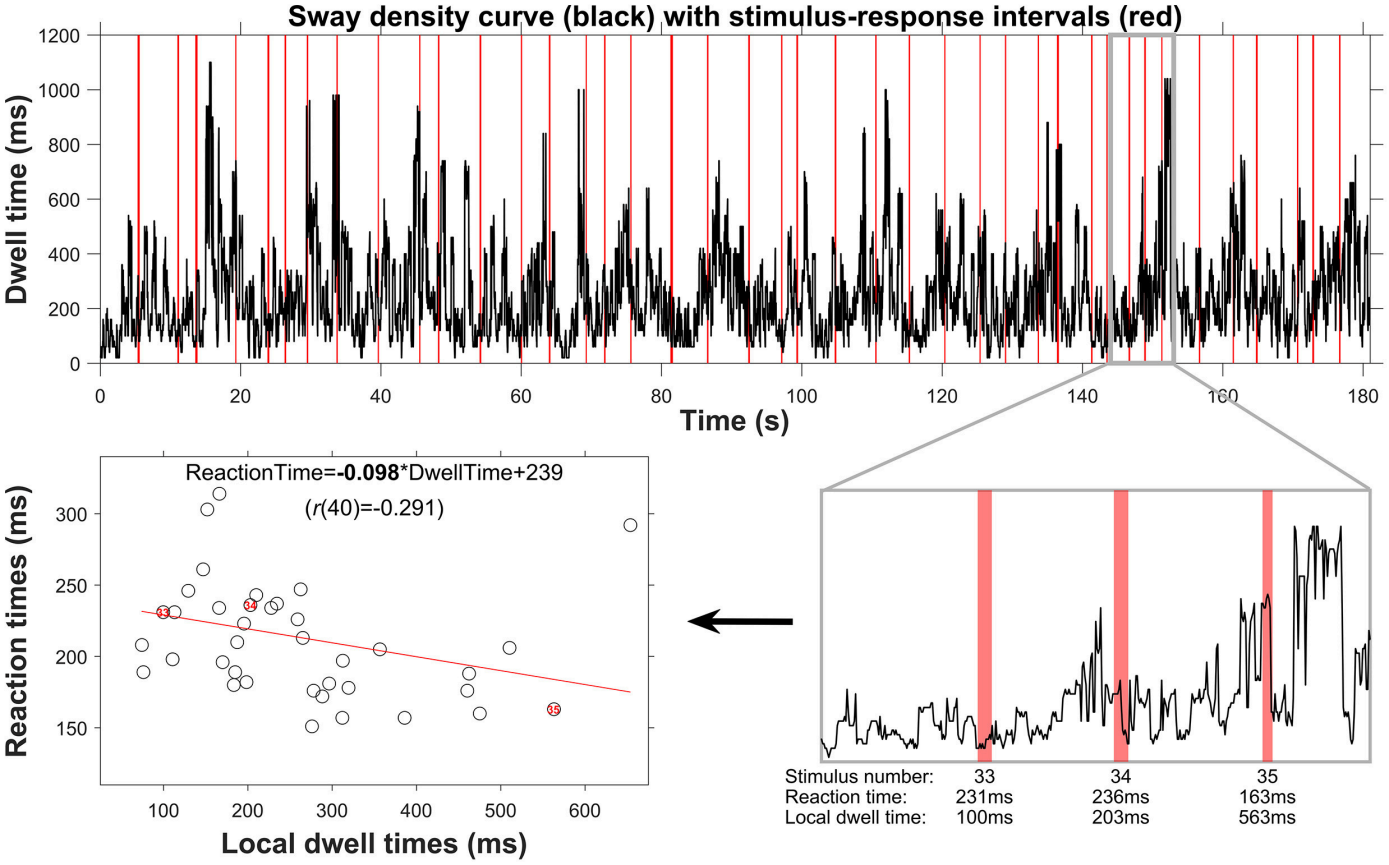
Procedure outlining the posturographic analyses from global to local dwell-time estimation, and their relation to stimulus-response reaction times. The top panel displays the sway density curve, with in red the 41 episodes corresponding to stimulus-response intervals. Over the whole trial, the dwell time was on average 248 ms, but dwell times clearly fluctuate throughout the trial, with peaks up to 1,000 ms. In the lower-right panel, the sway-density curve is depicted for a period of about 10 s, containing three stimulus-response pairs (numbers 33, 34, and 35). As can be seen, the local dwell times taken over the stimulus-response intervals, as well as the reaction-time values, vary. This is confirmed by the negative correlation between local dwell times and reaction times, as depicted in the lower-left panel, with the linear fit (red line) and its regression equation yielding an overall negative slope (presented in bold font) for this trial.

For each trial, Pearson's correlation coefficients and the slopes of the linear fits among these local posturographic values, as well as between local posturographic values and stimulus-response reaction times were determined and used for further statistical analyses (see below). The method of determining local dwell times and its association with stimulus-response reaction times (from which the slope was taken) is shown in Figure 2, for a representative trial (i.e., data corresponds to the left posturogram of Figure 1).

### Statistical analyses

We first tested the effect of surface (firm vs. foam) on stimulus-response reaction times and on the posturographic outcomes eccentricity and velocity. We performed two statistical analyses contrasting the two surface conditions, namely (1) a paired-samples *t*-test (alpha = 0.05) and (2) a Bayesian comparison of means (with default Cauchy prior of 0.707) performed in JASP (Version 0.8.6). The tests were performed on the average over the two trial repetitions per surface condition, except for one participant for which we used the second firm surface trial to represent the firm condition because of an error in data collection for the first firm trial. Bayesian hypothesis testing is rapidly gaining popularity [e.g., (10)]. It can be used to quantify the relative predictive value of two competing hypotheses, operationalized with so-called Bayes factors (BF) quantifying the relative evidence for the null hypothesis vis-à-vis the alternative hypothesis. The BF_01_ indicates how much more likely the data support the null-hypothesis (the means do not differ) compared to the alternative hypothesis (the means differ). BF_10_ equals 1/BF_01_, and quantifies how much more likely the data support the alternative hypothesis. It has been suggested to treat BFs between 1 and 3 as anecdotal (hence, inconclusive) evidence, BFs between 3 and 10 as moderate evidence, and BFs > 10 as strong evidence (11); we regard these qualifications as convenient shorthands to an underlying continuum of evidence.

With regard to the dwell times derived from the sway density analysis, we conducted a 2 (surface: firm, foam) by 10 (radius: 0.25, 0.50, …, 2.50 mm) repeated-measures ANOVA to (1) confirm the effects of magnitude of the posturogram and radius on the overall dwell times (smaller posturograms and larger radii would result in longer dwell times) and (2) to identify the radius yielding overall dwell times representative of the time scale of interest (i.e., dwell times similar to stimulus-response reaction-time intervals).

Third, we analyzed the statistical relationships among our local posturographic outcomes (local eccentricity, local velocity and local dwell times) as well as between these local outcomes and reaction times. We did this by analyzing the sign, magnitude and significance of the correlation coefficient as well as the slope of the linear fit. Since we were primarily interested in the nature and direction of the effect (positive, zero or negative), we entered the values of the correlation coefficient and the slope (averaged over the two trial repetitions) into one-sample *t*-tests against 0; correlations and slopes significantly different from zero would indicate an overall consistent positive or negative relationship. We likewise computed Bayes factors to quantify how much more likely the data supports the null hypothesis (correlations and slopes do not differ from 0, BF_01_) or the alternative hypothesis (correlations and slopes differ from 0; BF_10_). We did this separately for the firm and foam conditions.

## Results

### Reaction times

**Figure 3:**
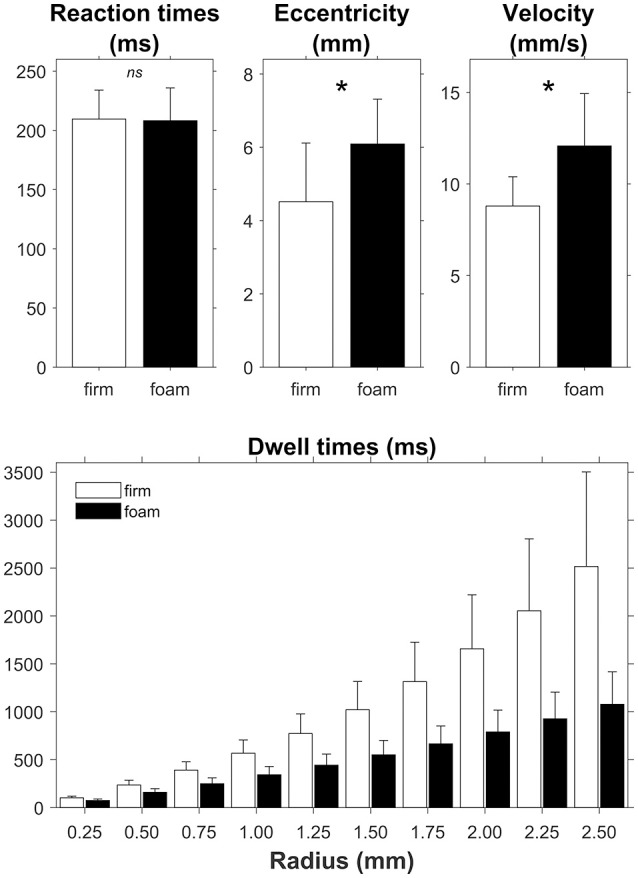
Effect of support surface (firm, white bars and foam, black bars) on reaction times and the global posturographic outcomes eccentricity, velocity and dwell times, the latter presented for a range of radius values. Error bars represent standard deviations. Asterisks in the top panels denote a significant difference between firm and foam conditions. The difference between firm and foam was significant across all radii (bottom panel).

For the RTs we found no significant difference between responding on a firm surface (210 ± 25 ms) and on a foam surface [208 ± 28 ms; *t*_(26)_ = 0.531, *p* = 0.60, *d* = 0.102; Figure 3, top left panel]. The Bayesian analysis yielded a BF_01_ of 4.31, thus indicating moderate evidence in favor of the null hypothesis, i.e., no effect of the foam manipulation on RT.

### Global posturography

Figure 3 further summarizes the effects of support surface on global posturographic outcomes. As predicted, standing on foam had very strong effects, in expected directions, with greater eccentricity, faster velocity and shorter dwell times on foam compared to standing on the firm surface. Specifically, significant effects were found for eccentricity [firm: 4.5 ± 1.6 mm, foam 6.1 ± 1.2 mm; *t*_(26)_ = −5.913, *p* < 0.001, *d* = −1.138; BF_10_ > 1,000) and velocity (firm: 8.8 ± 1.6 mm/s, foam: 12.1 ± 2.9 mm/s; *t*_(26)_ = −8.578, *p* < 0.001, *d* = −1.651; BF_10_ > 1,000). Furthermore, both surface and radius significantly affected the overall dwell time of the sway density analysis. That is, the 2 (surface: firm, foam) by 10 (radius: 0.25, …, 2.50 mm) repeated-measures ANOVA revealed shorter dwell times for foam (528 ms) than firm (1,063 ms) surfaces [main effect of Surface; *F*_(1, 26)_ = 89.77, *p* < 0.001, ES = 0.775]. Dwell times increased with increasing radii [main effect of radius; *F*_(9, 234)_ = 225.74, *p* < 0.001, ES = 0.897], with significant *post-hoc* differences between all radii (all *p*'s < 0.001). The interaction between surface and radius was also significant [*F*_(9, 234)_ = 72.42, *p* < 0.001, ES = 0.736], with significant between-surface differences growing with increasing radii (Figure 3, lower panel). Note that the average dwell times observed for a radius of 0.5 mm (198 ms) best corresponded to the average stimulus-response reaction times (209 ms), and hence to the time scale of interest. Consequently, we determined local dwell times based on a fixed radius value of 0.50 mm for the remainder of the analysis.

### Relationships among local posturographic outcomes

Table 1 shows the correlations, slopes and statistics among the three local posturographic outcomes: local eccentricity, local velocity and local dwell times. As can be seen, there were consistent associations among all local posturographic outcomes (i.e., correlations and slopes differed significantly from zero), with higher velocity at greater eccentricity, shorter dwell times at greater eccentricity and particularly shorter dwell times at higher velocity, for firm and foam surfaces alike.

**Table 1:** Mean slopes (slope), Pearson correlation coefficients (*r*), and their standard deviations (SD) as well as the number of significant positive (+) and negative (-) correlations of all trials *n* among local eccentricity, local velocity and local dwell times, separately for firm and foam surfaces.

		**Mean ±SD**	***t*_(26)_**	***p***	***d***	**BF_10_**
Eccentricity—velocity (firm)	slope	0.20 ± 0.30	3.496	0.002	0.673	21.3
	*r*	0.13 ± 0.17	3.935	< 0.001	0.757	57.4
	+/−/*n*	14/0/53				
Eccentricity—velocity (foam)	slope	0.15 ± 0.23	3.465	0.002	0.667	19.8
	*r*	0.10 ± 0.12	4.164	< 0.001	0.801	97.7
	+/−/*n*	7/0/54				
Eccentricity—dwell times (firm)	slope	−6.64 ± 8.46	−4.080	< 0.001	−0.785	80.3
	*r*	−0.11 ± 0.14	−4.023	< 0.001	−0.774	70.4
	+/−/*n*	1/7/53				
Eccentricity—dwell times (foam)	slope	−2.17 ± 3.01	−3.746	< 0.001	−0.721	37.3
	*r*	−0.09 ± 0.10	−4.271	< 0.001	−0.822	125.6
	+/−/*n*	0/3/54				
Velocity—dwell times (firm)	slope	−29.56 ± 11.71	−13.122	< 0.001	−2.525	>1,000
	*r*	−0.68 ± 0.06	−55.078	< 0.001	−10.600	>1,000
	+/−/*n*	0/53/53				
Velocity—dwell times (foam)	slope	−14.89 ± 7.65	−10.107	< 0.001	−1.945	>1,000
	*r*	−0.72 ± 0.05	−82.504	< 0.001	−15.878	>1,000
	+/−/*n*	0/54/54				

*Statistics pertain to the one-sample t-tests against 0, with Cohen's d as effect size. BF_10_ represents the Bayes factor, with values > 10 quantifying strong evidence for the alternative hypothesis (slope differs from zero) relative to the null hypothesis (slope does not differ from zero)*.

### Relationships between local posturographic outcomes and reaction times

Table 2 shows the correlations, slopes, and statistics between the three local posturographic outcomes and RT. While the correlation and slope for the relationship between local eccentricity and RT was not significantly different from zero, and anecdotal (BF_10_ between 1/3 and 1), the values of the correlations and of the slopes between RT and local velocity, and between RT and local dwell times, differed significantly from zero. The correlations were overall weak and mostly not significant at the level of a single trial. Nevertheless, as predicted, the relationship between velocity and RT was consistently positive, implying longer RTs for sway episodes of greater velocity. In addition, the association between local dwell times and RT was consistently negative, implying longer RTs for episodes with lower local dwell times. This set of results is in agreement with the abovementioned strong negative correlation between local velocity and local dwell times (Table 1). The significant effects (in terms of both frequentist and Bayesian analyses) point to highly consistent behavior across participants, for firm and foam surfaces alike.

**Table 2:** Mean slopes (slope), Pearson correlation coefficients (r), and their standard deviations (SD) as well as the number of significant positive (+) and negative (–) correlations of all trials *n* between the three local posturographic outcomes and reaction times (RT), separately for firm and foam surfaces.

		**Mean ±SD**	***t*_(26)_**	***P***	***d***	**BF_10_**
Eccentricity—RT, firm	slope	1.04 ± 3.01	1.801	0.083	0.347	0.8
	*r*	0.04 ± 0.16	1.342	0.191	0.258	0.4
	+/−/*n*	5/1/53				
Eccentricity—RT, foam	slope	0.46 ± 1.87	1.267	0.216	0.244	0.4
	*r*	0.04 ± 0.12	1.790	0.085	0.344	0.8
	+/−/*n*	5/0/54				
Velocity—RT, firm	slope	2.03 ± 2.18	4.843	< 0.001	0.932	487.9
	*r*	0.13 ± 0.12	5.516	< 0.001	1.062	>1000
	+/−/*n*	8/0/53				
Velocity—RT, foam	slope	0.82 ± 0.95	4.470	< 0.001	0.860	201.0
	*r*	0.11 ± 0.10	5.658	< 0.001	1.089	>1000
	+/−/*n*	7/0/54				
Dwell times—RT, firm	slope	−0.051 ± 0.073	−3.656	0.001	−0.704	30.4
	*r*	−0.10 ± 0.12	−4.633	< 0.001	−0.892	295.4
	+/−/*n*	1/4/53				
Dwell times—RT, foam	slope	−0.042 ± 0.050	−4.368	< 0.001	−0.841	157.8
	*r*	−0.07 ± 0.09	−4.077	< 0.001	−0.785	79.8
	+/−/*n*	0/3/54				

*Statistics pertain to the one-sample t-test against 0, with Cohen's d as effect size. BF_10_ represents the Bayes factor, with values > 10 quantifying strong evidence for the alternative hypothesis (slope differs from zero) relative to the null hypothesis (slope does not differ from zero)*.

## Discussion

The aim of this study was to test a key prediction that could be derived from the intermittent control theory of quiet upright standing. Based on biomechanical measurements it has been argued [e.g., (1)] that quiet standing is accomplished by a dual system, involving stiffness control, and feedforward control (based on anticipatory top-down regulation). Various papers (see Introduction) have emphasized that these control mechanisms seem to alternate in rapid succession, akin to eye movements consisting of saccades and fixations. We reasoned that passive stiffness control during quiet standing would be less computationally demanding than episodes involving “intermittent stabilization bursts” (9). In other words, the attentional load of maintaining stable upright stance was supposed to fluctuate during quiet standing, depending on which of the two control mechanisms happened to be at play. If this holds, then attentional load (as indexed using stimulus-response reaction time) should vary with local posturographic features reflecting active and passive control.

Our findings were as follows. First, we found that the mean reaction time did not differ between support surface conditions (i.e., firm vs. foam; Figure 3). A comparable study by Vuillerme and Nougier (12) found evidence for increased attentional requirements when standing on foam, but the effect (i.e., longer reaction times for foam than firm surfaces) was only found in a group of non-gymnasts, whereas gymnasts (with presumably superior balance abilities) showed no effect of standing on foam on RT. Our finding that RT did not differ between the two surface conditions suggests that our subjects had very good balance abilities, requiring very little attentional resources [as in (12)], and/or prioritized the reaction-time task over the balance task, focusing predominantly on RT performance, which is not unlikely given the observed posturographic changes when standing on foam (greater overall eccentricity, greater overall velocity and lower overall dwell time; Figure 3).

Second, when focusing exclusively on COP segments encompassing the stimulus-response intervals, we found a consistent statistical association among our three local posturographic features (Table 1). That is, we found that COP segments with relatively high local eccentricity (i.e., far removed from the center of the posturogram) also had relatively high local velocity and relatively low local dwell times. Although these correlations are relatively weak in magnitude and mostly not significant at the level of a single trial, the consistent positive and negative relationships over trials and participants reflect that the COP generally moves faster and dwells shorter, the further away it is from the origin, and vice versa. Such faster movements in more eccentric positions could signal an imminent loss of stability near the periphery (i.e., the onset of falling due to an accelerating center of mass) and/or postural adjustments to bring the eccentric center of mass back to the relative safety of the origin. Note that the negative correlation between local COP velocity and local dwell times was significant for all trials and quite strong in magnitude (Table 1), as it should be by definition.

Third, and this was the core finding of the study, we found consistent associations between attentional load (RT) and local velocity and local dwell times, but not between RT and postural eccentricity (Table 2). Participants generally took more time to respond to an auditory stimulus when their COP happened to move fast, and vice versa. Likewise, participants generally responded slower to the stimuli in COP episodes with lower dwell times (see also lower-left panel of Figure 2). In contrast, stimulus-response reaction times did not vary systematically with how far away from the origin the COP was. These main findings have several implications. To start with, the increase in RT with lower local dwell times and faster COP velocity is in line with our main prediction. It suggests that phases in the COP trajectory with high velocity and low dwell times are associated with an elevated attentional cost, presumably because these posturographic features reflect instances of active control. Comparable findings have been reported in the literature on rhythmic arm movements [e.g., (13–15)], where it is found that RT is slowed down in certain phases in the movement cycle, possibly due to attentional engagement during “anchoring” [i.e., discrete instances in the continuous movement cycle during which control is exerted over the oscillator; see also (16, 17)]. In the context of locomotion, Lajoie et al. (18) found that reaction times to an auditory stimulus also varied systematically over the gait cycle; RTs were higher during the single-support phase compared to the double-support phase. These findings suggest that classical cognitive-motor dual-tasking effects [e.g., (19)] are visible not only across groups and conditions (e.g., comparing single task to dual-task performance), but also within a single continuous movement trajectory, such as a COP trace or cyclic movements. Classical dual-tasking studies typically compute average performance scores of the component tasks across the entire measurement, to assess the overall attentional requirements of the dual task at various difficulty levels. Our approach, in contrast, revealed that the attentional requirements of the continuous motor task of quiet standing exhibit an “ebb and flow”, as evidenced by variations in reaction time that varied weakly but consistently with local posturographic features. This finding may help develop theorizing about the hypothesized dualistic nature of postural control, for example as regards the relative contribution of each mechanism and their respective time courses.

Two other implications follow from our main findings. First, the finding that local dwell times and local COP velocity are strongly interrelated (Table 1), while both correlate weakly but consistently with RT intervals (Table 2), implies that local COP velocity may yield the same information as local dwell times derived from sway density analysis. We would like to stress, however, that this redundancy only holds for our analysis approach where we correlated selected segments in the COP, and not necessarily applies to the entire time series. After all, several other parameters can be deduced from sway density curves (e.g., number of peaks, peak height, distance or time between peaks) that proved quite useful for between-task or between-group comparisons in the study of postural control [e.g., (7)]. If and when future studies find that, across different task conditions and populations, local COP velocity gives identical results as the local dwell times derived from sway density curves, then one might consider sticking to local COP velocity, as it is conceptually and computationally more straightforward. Second, our finding that RT was not associated with postural eccentricity in our sample of 27 young adults is in line with the original finding of Teasdale et al. (8), who found an effect only for older participants and not the young. It should be noted that their results were based on nine elderly and eight young, and that they did not employ Bayesian statistics to provide evidence in favor of the null. In combination with our results, it seems fair to conclude that the attentional demands of maintaining quiet upright standing in young adults are not related to eccentricity but instead to local velocity and local dwell time, regardless of their position in the posturogram.

All in all, our results support a key prediction from the intermittent control theory of quiet standing by showing longer RTs for episodes containing local posturographic features indicative of active control (i.e., lower local dwell times, higher local velocity). Although the slopes and correlation coefficients of these associations were consistently and convincingly different from zero, it must be stressed that the correlation values were overall relatively low (roughly between 0.1 and 0.2) and mostly not significant at the level of a single trial (Table 2). These low correlations could partly be explained by the adopted approach for stimuli presentation. That is, we presented stimuli at random instances during a quiet standing trial, and we later correlated reaction times to local posturographic features that happened to be present at that moment in time. As can be appreciated from Figure 2, our stimuli generally missed the brief episodes with very high dwell times, that is, the episodes where the COP is relatively stationary and assumed to reflect passive control. We further noticed that episodes with high dwell time (peaks in the sway density curve) are typically quite short in duration, and notably shorter than the duration of a typical stimulus-response event. Looking again at the dwell times over a representative trial shown in the top panel of Figure 2, one can clearly see that the peaks (representing little COP movement) are shorter in duration than the intermediate episodes with lower dwell times (faster COP movement). As a consequence, most stimulus events will occur during such episodes of high postural activity. The typical short duration of episodes with high dwell times also implies that local dwell time estimates may reflect a mixture of passive and active control, such as for stimulus 34 in Figure 2. These three factors all reduce the explained variance of RT as a function of local dwell time and local velocity, and may hence explain why, despite the overall consistent directional trends, the magnitude of the correlations tends to be relatively low.

The observed slowing of reaction time with fast postural adjustments could point to the presence of a refractory period. This notion is quite common in the psychological literature and states that responding to a second event is slowed down if the event is in close temporal proximity to an immediately preceding stimulus-response event. Information processing of the first event takes some time to complete, which interferes with processing the following event, as demonstraded by delayed reaction times. The notion of refractoriness has recently been applied to the field of motor control by a study of van de Kamp et al. (20). In that study subjects had to manually control an unstable (virtual) inverted pendulum using a continuous joystick task. The pendulums varied in stability and system order. It was found that stabilization of the pendulum could be described by a series of brief ballistic control episodes, instead of continuous control. According to the authors, their data suggest a refractory period during which open loop control is not possible due to a hypothesized single-channel processing bottleneck. Moreover, continuous control was also unnecessary, since intermittent (serial ballistic) control was capable of stabilizing the unstable system. It could very well be that refractoriness is a general physiological mechanism that operates to control a wide range of homeostatic systems, including maintaining upright standing posture. We speculate that our observation of longer reaction times with shorter dwell times is compatible with the notion of such a general (cross-modal) bottleneck. More specifically, such an active intermittent postural control episode causes a brief temporal interference (i.e., refractoriness), blocking further response processing, such as responding to the auditory stimulus.

In conclusion, by mapping stimulus-response intervals to local posturographic features we demonstrated attentional fluctuations in the control of quiet upright standing, thereby validating core assumptions underlying the sway-density analysis and the theoretical notion of intermittent postural control. Future studies are recommended to control the presentation of the stimuli in a movement-dependent manner, such as successfully done in the context of cyclic tasks (15, 21, 22), in order to increase the likelihood of obtaining episodes with high dwell times. This may also be instrumental in identifying the method (i.e., local dwell times or some other local posturographic parameter) that best parameterizes intermittent postural control, which seems relevant given the low magnitude of the correlations between local dwell times and reaction times. Furthermore, future studies are recommended to record EMG data of a set of muscles relevant to postural control. Complementing local posturographic features and associated stimulus-response intervals with recordings of neural activity could help unveil the neural circuitry driving the musculoskeletal control of posture (23–25). Moreover, when adopting an event-based approach, it could perhaps even pinpoint differences therein for the hitherto identified active and passive control regimes. To our knowledge, this has never been directly tested, despite offering a potentially informative window into the control processes underlying intermittency.

## Data availability statement

The raw data (Matlab struct containing all data) supporting the conclusions of this manuscript will be made available by the authors, without undue reservation, to any qualified researcher. Processed data, i.e., means, are available as Supplementary Material to this paper.

## Ethics statement

The study was approved by the local ethics committee of the Vrije Universiteit of Amsterdam. All subjects gave written informed consent in accordance with the Declaration of Helsinki.

## Author contributions

JS developed the study concept and research plan and conducted the research. MR and JS conducted the data analysis. JS and MR wrote the paper.

### Conflict of interest statement

The authors declare that the research was conducted in the absence of any commercial or financial relationships that could be construed as a potential conflict of interest.
